# Determinants of perceived quality of obstetric care in rural Tanzania: a cross-sectional study

**DOI:** 10.1186/1472-6963-14-483

**Published:** 2014-10-18

**Authors:** Elysia Larson, Sabrina Hermosilla, Angela Kimweri, Godfrey M Mbaruku, Margaret E Kruk

**Affiliations:** Health Policy & Management, Columbia University, 600 W. 168th St, New York, NY 10032 USA; Epidemiology, Columbia University, 722 W. 168th St, New York, NY 10032 USA; Ifakara Health Institute, Plot 463, Kiko Avenue Mikocheni, Dar es Salaam, Tanzania

**Keywords:** Quality of health care, Health services, Maternal health, Measurement

## Abstract

**Background:**

Patients’ reported opinions of the health system need to be understood in order to provide patient-centered care. We investigated determinants of women’s ratings of the quality of care during their most recent facility delivery.

**Methods:**

We conducted a census of all deliveries in the 6 weeks to 12 months preceding the survey, in villages served by 24 primary care clinics in rural Pwani Region, Tanzania. Women who had delivered children in a study facility were included in this analysis (n = 855). We interviewed women about demographic and obstetric factors and the quality of their obstetric care using a structured questionnaire. We created a composite index of perceived quality from six quality questions. We also assessed the functioning of the local health clinic using structured surveys. We used a multi-level model to analyze factors associated with women’s rating of the quality of care during delivery.

**Results:**

14% of respondents rated the overall quality of care received during delivery as excellent. Women who listened to the radio daily reported lower quality composite scores (β: -0.99, p < 0.001). Women who reported receiving more services in ANC had higher quality scores (β: 0.46, p = 0.001), as did women receiving more delivery services (β: 0.55, p < 0.001). Women who reported disrespect and abuse during delivery had significantly lower quality scores (β: -4.13, p < 0.001).

**Conclusions:**

A woman’s expectations and prior and current experiences influence her perception of the quality of care she received. Health facility characteristics did not influence ratings of overall quality. Focusing on improving the process rather than inputs of service delivery during ANC visits and delivery may increase perceived quality of delivery care in low-resource settings.

**Trial registration:**

ISRCTN17107760

## Background

Despite global reductions in under-five mortality and increasing life expectancies, large gaps remain in health progress, particularly in maternal and newborn health in sub-Saharan Africa [1]. The technical and financial capacity necessary to close these gaps exists at the global level, but implementation of known, efficacious approaches in low-resource settings has been slow [2, 3].

The maternal mortality ratio in Tanzania in 2013 was 390 maternal deaths per 100,000 live births [4]. Although only half of women utilize health facilities for delivery care nationally, some regions of Tanzania have seen dramatic increases in delivery care utilization. In Pwani region, in eastern Tanzania, facility deliveries increased from 43.0% in 2004 to 74.9% in 2010 [5, 6]. As utilization of health care increases, the spotlight is shifting to the quality of care provided in health facilities. Rapid response to obstetric emergencies is critical for saving the life of the mother and newborn [4]. But in Tanzania many designated delivery facilities are unable to provide basic emergency obstetric and newborn care (BEmONC) due to shortages of essential infrastructure, equipment, medicines, and above all, human resources [7–9].

The Tanzanian Ministry of Health, as well as numerous non-governmental organizations, are monitoring indicators of quality of care and working to implement improvements.

While objective measures of quality are important, so is understanding how health system users judge quality of care. This is because perceived quality is linked to future health care utilization decisions and overall trust in the health system [10, 11]. In addition, as the beneficiaries of care, patients are key stakeholders in the health system and their feedback is necessary to develop responsive, people-centered health systems. High quality ratings by patients can be seen as an indicator of a health system that is responsive to patient expectations.

While patient satisfaction indicators have been used as measures of the performance of the health system, a growing body of literature demonstrates that significant variation in patient satisfaction is due to patient characteristics, rather than features of the health system [12, 13]. Less is known about how patients judge the quality of care they have received and most prior studies have been qualitative [14]. In this study we explored factors associated with women’s rating of quality of care of their last obstetric visit. We use a unique dataset of facility characteristics and women’s self-reported data in order to assess the contributions of patients’ expectations and experiences on their perception of quality of care.

## Methods

### Study design and population

This analysis utilized baseline survey data collected for a cluster-randomized study of a quality improvement intervention for maternal and newborn health in four districts of Pwani Region, Tanzania: Bagamoyo, Kibaha Rural, Kisarawe, and Mkuranga (ISRCTN17107760). Pwani region is a primarily rural region in eastern Tanzania, with most of the population employed in small-scale subsistence farming or unskilled manual labor [6]. The Ministry of Health assigns each village to a primary health care facility called a dispensary. Dispensaries offer outpatient services including reproductive and child health services and uncomplicated deliveries. The dispensaries included in this study were staffed by medical attendants, nurses, and clinical officers. Health centers and hospitals are the next two tiers of healthcare in Tanzania and both offer inpatient and outpatient services. District and regional hospitals, as well as some health centers, offer comprehensive emergency obstetric and newborn care, including caesareans and blood transfusions. Health centers and hospitals both serve as referral centers for the lower-level health facilities [8, 15].

**Figure 1:**
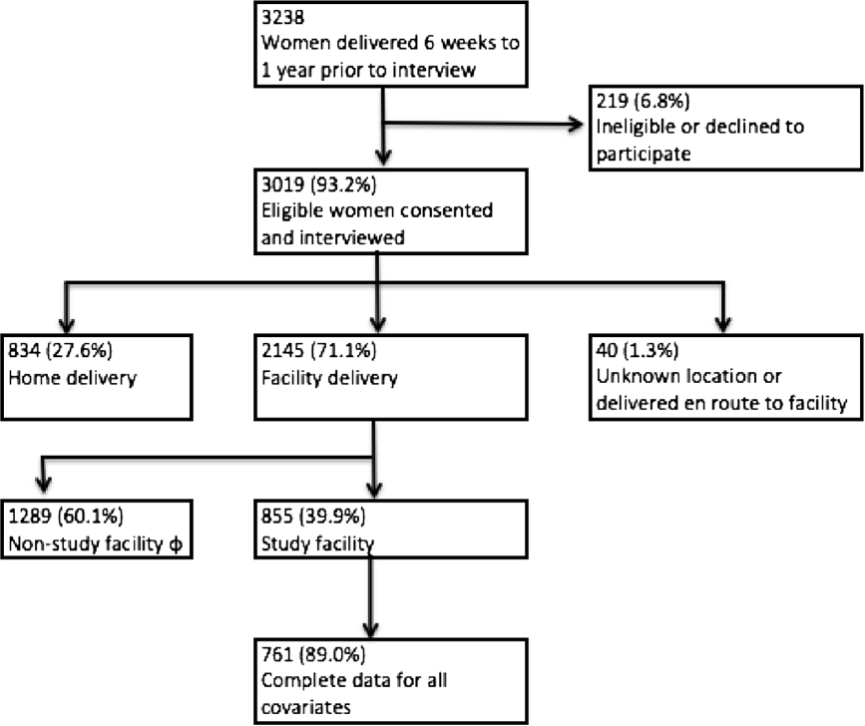
**Study population, Tanzania, 2012.** ϕ Of the women who delivered in a non-study facility, 59.8% delivered in a hospital, 18.5% delivered in a health center, and 21.7% delivered in a dispensary.

The study sites are 24 study dispensaries and the villages served by those facilities (i.e. villages officially designated to be in the facility’s catchment area). Facilities in the four districts were eligible for the study if they were government-managed primary care facilities, with at least one medically trained staff member (e.g. doctor, clinical officer, or nurse), were actively providing delivery services, and did not have an additional, ongoing large maternal and newborn health quality improvement project. From these, the six dispensaries with the highest volumes of deliveries were selected for inclusion in each district. The population-based survey was conducted between February 13 and April 28, 2012 as part of the baseline assessment for the study.We conducted a full census of all households in the study areas (30,076 households) to identify women who delivered between six weeks and one year prior to interview and were at least 15 years of age. Identified women were invited to participate in a structured interview. All women who provided written consent, or in the case of minors under 18 years of age, their assent and guardian consent, were interviewed. Participants were included in the current analysis if they delivered their most recent child in one of the 24 facilities included in the study (Figure 1).

The survey and consents were developed in English, translated to Swahili, back-translated and pre-tested to ensure accuracy. Detailed data collection methods have been previously reported [16].

We also conducted an assessment of the 24 primary care facilities serving the study population from December 5, 2011 to May 15, 2012 utilizing a structured questionnaire that assessed human resources, infrastructure, and services available at the facility. The survey was adapted from the needs assessment created by the Averting Maternal Death and Disability Program (AMDD) and the UN system that has been previously used in more than 30 countries, including Tanzania [17]. During the same time period we administered structured job satisfaction surveys to all health workers and clinical vignettes to all health workers trained to provide deliveries (nurses and clinical officers).

The study was approved by the ethical review boards at Columbia University in New York and in Tanzania by the Ifakara Health Institute and the Tanzanian National Institute for Medical Research.

### Measures

The outcome measure, patient-perceived quality of care, was created from women’s responses to six questions regarding aspects of the quality of care of their most recent delivery. These questions assess technical and non-technical aspects of quality of care [18]. Respondents rated each element of care using a five-level likert scale (excellent, very good, good, fair, poor). The scores were added to create a summative composite index with possible range from 6–30. We calculated Cronbach’s α to asses internal consistency of the scale. The questions were: (1) During your delivery, how would you rate your experience of being greeted and talked to respectfully? (2) How would you rate the knowledge and competence of health workers at this facility for this delivery? (3) During your delivery, how would you rate the experience of how clearly health care providers explained things to you? (4) During your delivery, how would you rate the cleanliness of the rooms inside the facility, including toilets? (5) How would you rate the quality of the drugs and modern equipment available at this facility (where you delivered)? (6) During your delivery, how would you rate the privacy you were given?

**Figure 2:**
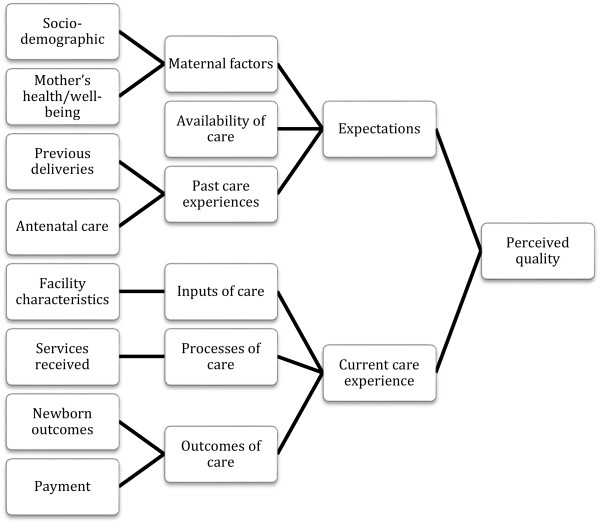
**Conceptual framework for the determinants of perceived quality of care.**

Our conceptual framework is informed by prior research that showed that patients’ perception of the quality of care received is dependent on both their expectations of care and the content of their care (Figure 2) [12, 14, 19]. Patients’ expectations in turn may be influenced by their social and economic standing, education, past health care experiences, self-perceived health and well-being and the opinions of their community members [13]. We assessed demographic and household variables including age, education (any secondary education versus less education as women with secondary education have been shown to be more selective users of health care) [20], occupation (farmer or homemaker versus skilled or student), woman as head of household as a measure of greater control of own health decisions, and exposure to radio (at least once per week versus less) as a measure of exposure to media. We also assessed each woman’s current health status using the EQ-5D (EuroQol Group, Rotterdam, Netherlands) as a measure of her general health status, expecting that women who are more ill may have higher expectations for health care than healthier women [11]. For each woman, we constructed an index of relative wealth using principal components analysis of a set of 18 questions on ownership of household assets [21]. We compared women in the top 20% of wealth against all other women, as wealthier women may have greater means to seek good health care and this in turn may shape their expectations of care in their local facility.

A woman’s past experiences and prior exposure to the health system were measured through her number of prior deliveries, if she had at least one prior delivery in a health facility (as a measure of her exposure to institutional delivery care), if she had ever had a child who died as a newborn (as a measure of a strong negative prior experience), if she attended antenatal care (ANC) for more than 3 visits for the delivery under study (as a measure of the intensity of her recent exposure to maternal health care), and the services she reported receiving in ANC (a standardized index of 8 items: weighed, height, blood pressure, urine sample, blood sample, malaria prophylaxis, tetanus vaccine, and iron supplements). In our study area, most women who deliver in their local dispensary also receive ANC there.

Following Donabedian’s model, we assessed the structure and process of care using facility data and maternal self-report [12]. To assess the structure, or inputs, we evaluated whether the facility had access to clean water, the number of health workers available, and the facility average health worker self-assessed confidence in obstetric, newborn, and HIV skills. We assessed health worker scores on clinical vignettes as a measure of the knowledge and competence of facility staff [22]. We developed an index of essential maternal and newborn equipment, supplies, and drugs using the Tanzanian Ministry of Health required list, previously reported indices, and an expert review panel [8, 23]. Structural indicators were measured at a single point in time between December 5, 2011 and May 15, 2012, but these are slow-changing and so likely prevailed during her delivery.

The process indicators at the facility are proxies for the services she may have received and include a standardized index of basic emergency obstetric functions provided in the past three months and a standardized index of the number postnatal services routinely provided (11 services included). We assessed the content of care the woman received during her delivery using an additive index of self-reported receipt of nine recommended services during delivery and immediately postpartum. These services were: mother checked, baby checked, uterotonic received, and mother given advice on: immediate feeding, exclusive breastfeeding, umbilical cord care, washing hands, immunization, and how to avoid chilling the baby. Other delivery variables included report of delivery complication and report of any disrespect or abuse. Women were asked if they experienced disrespect or abuse during delivery, and the terms were not further defined. The report is therefore their perception of what it means to experience disrespect or abuse. Outcomes of the visit included loans or sales of assets to pay for the delivery and child survival. Financial hardship related to delivery may negatively influence her rating of quality.

### Data analysis

Univariate statistics were calculated for individual and clinic-level characteristics and data were examined for variable distribution, outliers, and missingness. As noted above, covariates were categorized based on our conceptual framework (i.e., factors that influence expectations, secondary education, high wealth quintile). We standardized all indices in order to aid in interpretation of the regression model.

We conducted bivariate regressions with each potential covariate and the perceived quality index to guide the decision of which covariates would be included in the final multi-variable model. In order to assess the contributions of both individual and facility factors to women’s rating of quality we estimated three separate 2-level linear random intercept models: a null model without covariates, a model including individual-factors (and a district fixed effect), and a final model including individual, facility, and district covariates. We then calculated the proportion of the variation in perceived quality that was due to individual and random effects versus facility-level effects.

All analyses were conducted using Stata 12.1 (StataCorp LP, College Station, USA).

## Results

We conducted interviews with 3,019 of 3,238 eligible women (response rate: 93%). Of the interviewed women, 2,145 (71%) delivered their most recent child in a health facility; 855 (40%) of whom delivered their most recent child in a study facility and were therefore included in this analysis (Figure 1).

**Figure 3:**
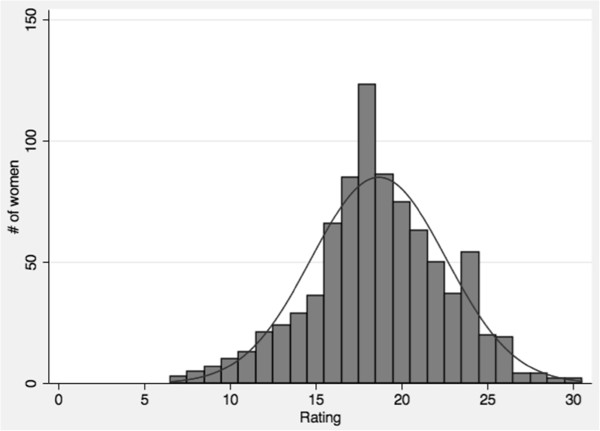
**Distribution of composite perceived quality index, created from ratings of six aspects of technical and non-technical quality of care, Tanzania, 2012.**

Characteristics of the 855 women and the 24 health facilities are reported in Table 1. Among study participants, 761 (89%) had complete information for all covariates of interest in the final adjusted model. The quality composite score was normally distributed with a Cronbach’s α of 0.81 (Figure 3). The average quality rating was 18.65 (range: 7–30) and 116 women (14%) rated the overall quality of care received during their delivery as excellent.

**Table 1:** **Descriptive statistics for full sample of 855 women, Tanzania 2012**

		n (%)
**Level 1: individuals (n = 855** ^**a**^ **)**		
**Maternal factors**		
**Socio-demographic**		
	Age (mean, SD)	27.66 ± 6.53
	Any secondary education	71 (8.31)
	Household wealth: richest 20%	188 (22.09)
	Farmer or homemaker	687 (81.01)
	Head of household	36 (4.21)
	Owns own mobile phone	319 (37.49)
	Listens to the radio daily	534 (62.46)
**Health/well-being**		
	Full function on EQ-5D^b^	589 (68.89)
**Availability of care**		
	Distance from village center to nearest hospital (km) (mean, SD)	39.19 ± 21.69
	Density of health facilities within 30 km of home village center (mean, SD)	13.74 ± 8.46
**Past maternal health experiences**		
	Number of previous deliveries (mean, SD)	2.45 ± 2.01
	Had at least one newborn who died	46 (5.38)
	>3 ANC visits for most recent pregnancy	532 (63.11)
	Number of ANC services received (max. 9)^c^ (mean, SD)	6.96 ± 1.56
**Current care experience**		
**Processes**		
	Number of delivery services received (max. 9)^d^ (mean, SD)	6.13 ± 2.22
	Experienced disrespect and abuse	77 (9.07)
	Had a complication during delivery	800 (94.12)
**Outcomes**		
	Child is alive	843 (98.83)
	Doctor/nurse fees, drugs, supplies, tests (USD) (mean, SD)	5.94 ± 4.84
	Borrowed money or sold assets to pay for services	105 (12.46)
**Dependent variable: quality ratings** ^**i**^		
	Greeted and talked to respectfully	322 (37.93)
	Knowledge and competence of health workers	301 (35.50)
	How clearly health care providers explained care	279 (32.86)
	Cleanliness of facility	223 (26.33)
	Availability of drugs and modern equipment	183 (21.73)
	Privacy	312 (36.79)
	**Level 2: facilities (n = 24)**	
**Current care experience**		
**Inputs/infrastructure**		
	Index of equipment, supplies, and drugs available (max. 29)^e^ (mean, SD)	16.38 ± 3.37
	Clean water	6 (25.00)
	Total health workers (mean, SD)	4.13 ± 1.65
	Facility average health worker confidence on 35 maternal, newborn, and HIV skills (mean, SD)	42.06 ± 20.72
	Health worker obstetric knowledge & competence score (mean, SD)^f^	41.40 ± 11.38
**Processes**		
	Number of emergency obstetric services provided in past 3 months (max. 7) (mean, SD)^g^	2.13 ± 1.15
	Number of postnatal services routinely provided (max. 11)^h^ (mean, SD)	7.96 ± 2.07
	Monthly facility deliveries in 2011 (mean, SD)	7.37 ± 4.90

± is mean SD.

^a^Values may not add up to 100 due to missing data.

^b^Woman reported highest level of health on all five EQ-5D questions.

^c^Index of 9 ANC services: diagnostics, treatment, and counseling.

^d^Index of 9 delivery and postnatal counseling services.

^e^Index of facility availability of 29 equipment, drugs, and supplies essential for maternal and neonatal care.

^f^Measured using clinical vignettes. Used as a proxy for services delivered.

^g^Index of 7 basic emergency obstetric and newborn (BEmONC) services performed in past 3 months.

^h^Index of 11 postnatal exam and services that the facility routinely provides for the mother and newborn.

^i^Proportion of women rating the quality as ‘excellent’ or ‘very good’ on a 5-point likert scale ranging from ‘poor’ to ‘excellent’. Dependent variable is a summative index of these six questions.

Maternal characteristics that affected the rating of quality of care were education and media exposure. Listening to the radio had a negative impact on women’s rating (β: -0.99, 95% CI: -1.52, -0.47). Of women’s past experiences, the only one affecting her quality rating was the number of services received during ANC; women who had received a greater number of ANC services rated the quality of delivery care higher than those who received fewer services (β: 0.46, 95% CI: 0.18, 0.74).

Structural facility indicators, such as having clean water or having essential equipment, drugs, and supplies were not associated with higher ratings of quality of care. Multiple factors describing the process of delivery care were associated with women’s ratings, including the number of delivery services she received, the number of postnatal services the facility routinely provides, and whether or not she experienced disrespect or abuse during delivery (Table 2).

**Table 2:** **Results of multilevel linear regression of associations between perceived quality and patient and facility-level characteristics, Tanzania, 2012**

	OLS coefficient (95% CI)
**Fixed effects: individual-level variables (n = 761)**	
**Maternal factors**	
Age	0.01 (-0.02, 0.05)
Any secondary education	-1.44 (-2.38, -0.51)**
Household wealth: richest 20%	-0.23 (-0.90, 0.44)
Listens to the radio daily	-0.99 (-1.52, -0.47)***
**Past maternal health experiences**	
Number of ANC services received^a^	0.46 (0.18, 0.74)**
Had at least one newborn who died	0.87 (-0.28, 2.02)
**Current care experience**	
**Processes**	
Number of delivery services received^b^	0.55 (0.27, 0.83)***
Experienced disrespect or abuse	-4.13 (-5.02, -3.24)***
**Outcomes**	
Borrowed money or sold assets to pay for services	-0.24 (-1.02, 0.53)
**Fixed effects: health facility-level variables (n = 24)**	
**Inputs/infrastructure**	
Index of equipment, supplies, and drugs available^c^	-0.10 (-0.50, 0.30)
Facility average health worker confidence on 35 maternal, newborn, and HIV skills	-0.03 (-0.05, -0.01)**
**Processes**	
Number of emergency obstetric services provided in past 3 months (max. 7)^d^	-0.07 (-0.36, 0.22)
Number of postnatal services routinely provided (max. 11)^e^	0.48 (0.15, 0.81)**
**Random effects (variance partitioned)**	
Individual-level plus random	100
Health facility-level	0

*P < 0.05; **P < 0.01; ***P < 0.001.

^a^Standardized index of 9 ANC services: diagnostics, treatment, and counseling.

^b^Standardized index of 9 delivery and postnatal counseling services.

^c^Standardized index of facility availability of 29 equipment, drugs, and supplies essential for maternal and neonatal care.

^d^Standardized index of 7 basic emergency obstetric and newborn (BEmONC) services performed in past 3 months.

^e^Standardized index of 11 postnatal exam and services that the facility routinely provides for the mother and newborn.

## Discussion

This study combined information on women’s characteristics and birth experiences with features of the facility at which she delivered, in order to explore how women’s expectations and experiences during the visit in question influence their ratings of the quality of delivery care. Such information can be used to improve quality of care and health systems responsiveness, pre-requisites for accountable health systems [24].

Informed by Donabedian’s quality model [12] and Sofaer and Firminger’s model of patient perceptions of quality [14], we developed a conceptual framework that included two main areas that may affect women’s reporting of quality: her expectations of care (which are informed by her socio-demographic characteristics as well as her prior experiences) and her experiences during the visit in question (inputs, processes and outcomes of care).

Women who had completed some secondary school and women who had higher media exposure rated the quality of delivery care lower than women with less education and media exposure. These women may have higher expectations of the care they should receive. Others have suggested that exposure to the media, which frequently tackles issues of government performance and problems with health care, may predispose women to rate care more negatively [13, 25].

In terms of women’s prior health care experiences, the only one associated with her rating of quality of delivery care was the number of services received during her ANC visit. Others have found that the quality of services received during ANC affect women’s utilization decisions for delivery [26, 27]. The local availability of health facilities, measured both by the distance from a woman’s hamlet to the nearest hospital and by the density of healthcare facilities within 30 km of her hamlet, did not affect her rating of the quality of care. This analysis was restricted to women delivering in primary care clinics, and as such cannot assess the role of proximity on quality ratings when women are traveling further distances and to access higher-level clinics. While prior studies have identified an association between parity (number of prior deliveries) and utilization of facilities for delivery [20, 27], we did not see an association between a woman’s parity and her rating of quality of care. This may be because the dependent variable in this study—a composite measure of care quality—referred specifically to the last delivery, and thus prior parity played a lesser role in the respondents’ rating.

Turning to the woman’s current delivery experience, most structural components (inputs and infrastructure) of the health facility generally did not influence quality ratings. Despite prior studies showing availability of drugs and supplies being a top driver of women’s preference for delivery facility, women in fact may have difficulty judging the availability of essential medications for acute care [28, 29]. We found that health worker confidence had a small negative effect on quality ratings while health worker scores on clinical vignettes had no effect. One hypothesis is that health worker confidence may be perceived by women as arrogance, or over-confident health workers may work quickly or communicate poorly with the patient. Past research showed that women’s rating of how well providers explained care was strongly correlated with the woman’s overall rating of quality, further supporting the importance of non-technical aspects of care [29]. We may not have seen an association between the health-care provider clinical vignette scores and woman’s ratings of quality in part because the clinical vignettes were only conducted with providers who have received formal training in basic emergency obstetric and newborn care [30], such as nurses and clinical officers, and other providers, such as maternal health aides, also conduct deliveries in some of the study facilities. Because women were not able to report the cadre of the health worker providing their obstetric services, we were not able to assess how perceived quality of care varied by cadre of health worker.

However, as expected, women’s experiences during the delivery had a strong impact on her rating of quality. Women who reported experiencing any disrespect or abuse from health workers during their visit reported quality ratings that were on average 4.1 points lower than women who did not report disrespect or abuse. Disrespectful treatment in facilities has been found to be distressingly common and has recently received substantial attention within the maternal health community as an abrogation of patient rights and an impediment to facility delivery [31, 32]. Experiencing a complication during labor and delivery, as reported by the woman, was not associated with her rating of quality of care. The vast majority (94%) reported a complication and thus this likely includes a range of adverse experiences in labor (i.e., pain) rather than actual medical complications. Further, it is likely that the nature of the health workers’ response to a complication, rather than the complication itself, would determine a woman’s rating of quality. It was not possible to measure the former in this study.

A greater number of clinical services received during labor and delivery improved women’s quality ratings, suggesting that women appreciate the importance of clinical procedures during delivery. We assessed two facility-level indicators of clinical processes: number of clinical services typically provided in the postpartum period and emergency obstetric services in the past three months. Providing a greater number of postpartum services was associated with an increased quality rating by the woman. Routine postpartum services reported by the facility are likely indicators of the services received by most women delivering there. There was no association between the number of emergency obstetric signal functions performed in the past three months and perceived quality, as facilities’ emergency capacity may not be visible to the vast proportion of women with uncomplicated deliveries.

Finally, the outcomes of delivery, costs of delivery and the survival of the child, did not affect quality ratings. The effect of the latter may be difficult to assess given its rarity: 1.2% of women in the full sample reported a newborn death.

This study has several limitations. We relied on self-report for specific services received, which may have introduced recall bias. However we expect this bias to be non-differential and thus would cause our results to be conservative. In future studies, matching observations of care by trained clinical observers to woman’s ratings may provide more accurate measures of association between perceived quality of care and the objective quality of services received. In addition, all our facilities were government-run, primary care clinics so there may not be a large variation in the features of health facilities serving the women in our sample, limiting our ability to detect health system features that impact quality. Because health centers and hospitals are expected to provider a wider range of maternal health services, including these facilities in future studies may provide a wider range of health system characteristics [33].

## Conclusions

Improving maternal and newborn health outcomes requires widespread utilization of high quality obstetric care. Improving the process of service delivery rather than focusing primarily on inputs may increase both objective and perceived quality of care. Attention to quality will be particularly important as women’s expectations continue rise with expanding education and exposure to media. Facility managers and providers should thus enhance the scope and appropriateness of antenatal and obstetric services provided, and ensure that these are delivered in a humane and respectful manner to every laboring woman.

## References

[1] Lozano R, Naghavi M, Foreman K, Lim S, Shibuya K, Aboyans V, Abraham J, Adair T, Aggarwai R, Ahn SY, Alvarado M, Anderson HR, Anderson LM, Andrews KG, Atkinson C, Baddour LM, Barker-Collo S, Barteis DH, Bell ML, Benjamin EJ, Bennett D, Bhalla K, Bikbov B, Bin Abduhak A, Birbeck G, Blyth F, Bolliger I, Boufous S, Bucello C, Burch M (2012). Global and regional mortality from 235 causes of death for 20 age groups in 1990 and 2010: a systematic analysis for the global burden of disease study 2010. Lancet.

[2] Jamison DT, Summers LH, Alleyne G, Arrow KJ, Berkley S, Binagwaho A, Bustreo F, Evans D, Feachem RG, Frenk J, Ghosh G, Goldie SJ, Guo Y, Gupta S, Horton R, Kruk ME, Mahmoud A, Mohohlo LK, Ncube M, Pablos-Mendez A, Reddy KS, Saxenian H, Soucat A, Ulltveit-Moe KH, Yamey G (2013). Global health 2035: a world converging within a generation. Lancet.

[3] Ronsmans C, Graham WJ (2006). Lancet maternal survival series steering g. Maternal mortality: who, when, where, and why. Lancet.

[4] Kassebaum NJ, Bertozzi-Villa A, Coggeshall MS, Shackelford KA, Steiner C, Heuton KR, Gonzalez-Medina D, Barber R, Huynh C, Dicker D, Templin T, Wolock TM, Ozgoren AA, Abd-Allah F, Abera SF, Achoki T, Adelekan A, Ademi Z, Adou AK, Adsuar JC, Agardh EE, Akena D, Alasfoor D, Alemu ZA, Alfonso-Cristancho R, Alhabib S, Ali R, Al Kahbouri MJ, Alla F, Allen PJ (2014). Global, regional, and national levels and causes of maternal mortality during 1990–2013: a systematic analysis for the global burden of disease study 2013. Lancet.

[5] National Bureau of Statistics, Tanzania & ORC Macro (2005). Tanzania Demographic and Health Survey 2004–05.

[6] National Bureau of Statistics, Tanzania & ORC Macro (2011). Tanzania Demographic and Health Survey 2010.

[7] Hsia RY, Mbembati NA, Macfarlane S, Kruk ME (2012). Access to emergency and surgical care in sub-Saharan Africa: the infrastructure gap. Health Policy Plan.

[8] Tanzania. Reproductive and Child Health Section (2008). The National Road map Strategic Plan to Accelerate Reduction of Maternal, Newborn, and Child Deaths in Tanzania, 2008–2015.

[9] Kurowski C, Wyss K, Abdulla S, Mills A (2007). Scaling up priority health interventions in Tanzania: the human resources challenge. Health Policy Plan.

[10] Andaleeb SS (2001). Service quality perceptions and patient satisfaction: a study of hospitals in a developing country. Soc Sci Med.

[11] Gilson L (2003). Trust and the development of health care as a social institution. Soc Sci Med.

[12] Donabedian A (2005). Evaluating the quality of medical care. 1966. Milbank Q.

[13] Bleich SN, Ozaltin E, Murray CK (2009). How does satisfaction with the health-care system relate to patient experience?. Bull World Health Organ.

[14] Sofaer S, Firminger K (2005). Patient perceptions of the quality of health services. Annu Rev Public Health.

[15] Tanzania., Tanzania. Wizara ya Afya (2003). National Health Policy.

[16] Kruk ME, Hermosilla S, Larson E, Mbaruku GM (2014). Bypassing primary care clinics for childbirth: a cross-sectional study in the Pwani region, United Republic of Tanzania. Bull World Health Organ.

[17] Paxton A, Bailey P, Lobis S, Fry D (2006). Global patterns in availability of emergency obstetric care. Int J Gynaecol Obstet.

[18] Valentine N, Darby C, Bonsel GJ (2008). Which aspects of non-clinical quality of care are most important? Results from WHO’s general population surveys of “health systems responsiveness” in 41 countries. Soc Sci Med.

[19] Andersen RM (1995). Revisiting the behavioral model and access to medical care: does it matter?. J Health Soc Behav.

[20] Abeje G, Azage M, Setegn T (2014). Factors associated with Institutional delivery service utilization among mothers in Bahir Dar City administration, Amhara region: a community based cross sectional study. Reprod Health.

[21] Filmer D, Pritchett LH (2001). Estimating wealth effects without expenditure data - or tears: an application to educational enrollments in states of India. Demography.

[22] Peabody JW, Luck J, Glassman P, Jain S, Hansen J, Spell M, Lee M (2004). Measuring the quality of physician practice by using clinical vignettes: a prospective validation study. Ann Intern Med.

[23] Keyes EBP, Shelus V, Pearson L (2013). Measuring Facility Readiness to Deliver Emergency Obstetric and Newborn Care (EmONC).

[24] Rockers PC, Kruk ME, Laugesen MJ (2012). Perceptions of the health system and public trust in government in low- and middle-income countries: evidence from the World Health Surveys. J Health Polit Policy Law.

[25] Judge K, Solomon M, Miller D, Philo G (1992). Public opinion, the NHS, and the media: changing patterns and perspectives. BMJ.

[26] Barber S (2006). Does the quality of prenatal care matter in promoting skilled institutional delivery? A study in rural Mexico. Matern Child Health J.

[27] Rockers PC, Wilson ML, Mbaruku G, Kruk ME (2009). Source of antenatal care influences facility delivery in rural Tanzania: a population-based study. Matern Child Health J.

[28] Hanson K, McPake B, Nakamba P, Archard L (2005). Preferences for hospital quality in Zambia: results from a discrete choice experiment. Health Econ.

[29] Kruk ME, Paczkowski M, Mbaruku G, de Pinho H, Galea S (2009). Women’s preferences for place of delivery in rural Tanzania: a population-based discrete choice experiment. Am J Public Health.

[30] Lobis S, Mbaruku G, Kamwendo F, McAuliff E, Austin J, De Pinho H (2011). Expected to deliver: alignment of regulation, training, and actual performance of emergency obstetric care providers in Malawi and Tanzania. Int J Gynaecol Obstet.

[31] Bowser D, Hill K (2010). Exploring Evidence for Disrespect and Abuse in Facility-Based Childbirth; Report of a Landscape Analysis.

[32] Kruk ME, Kujawski S, Mbaruku G, Ramsey K, Moyo W, Feedman L (2014). Disrespectful and abusive treatment during facility delivery in Tanzania: a facility and community survey. Health Pol Plan.

[33] IHI (2013). Tanzania Service Availability and Readiness Assessment (SARA) 2012.

[CR34] The pre-publication history for this paper can be accessed here:http://www.biomedcentral.com/1472-6963/14/483/prepub

